# Analysis of Smoking Behavior in Patients With Peritonsillar Abscess: A Rural Community Hospital's Experience

**DOI:** 10.7759/cureus.23300

**Published:** 2022-03-18

**Authors:** Chelsea Clark, Anthony Santarelli, Stefan Merrill, John Ashurst

**Affiliations:** 1 Emergency Medicine, Texas College of Osteopathic Medicine, Fort Worth, USA; 2 Emergency Medicine, Kingman Regional Medical Center, Kingman, USA

**Keywords:** rural emergency medicine, smoking tobacco, tobacco abuse, peritonsillar abscess, emergency medicine

## Abstract

Background

Peritonsillar abscesses (PTA) are the most common deep space infection of the head and neck. They appear to have an association with a patient’s smoking history but data showing this relationship is sparse and controversial. Currently, no data on this association exists for those who seek care at a rural community emergency department (ED). Based upon the lack of data in this setting, the authors sought to determine the incidence, treatments, and outcomes between smokers and non-smokers with a PTA at a rural community ED.

Methods

A retrospective chart review of all patients undergoing a soft tissue neck computed tomography (CT) scan with or without intravenous contrast was completed from September 25th, 2019 through October 4th, 2021. Patients with a previously diagnosed PTA and those diagnosed via another means (clinical, needle aspiration, etc.), or outside of the ED were excluded from the dataset. Abstracted data included demographics, treatments, and outcomes of each patient. The data were analyzed using the Mann-Whitney test for continuous data and the chi-square test for categorical data.

Results

During the study period, a total of 50 patients were diagnosed with a PTA via soft tissue neck CT. Of those diagnosed, the median age was 40.5 (25.5 - 53.3) years, 15 were female, 38 self-identified as white, and 27 noted a current smoking history. Smokers presented to ED earlier than non-smokers (2.0 vs 4.0 days; p=0.03), but no difference was noted in the size of PTA identified via CT (2.0 vs 1.5 cm; p=0.13). No difference among smokers and non-smokers was noted in corticosteroid therapy either administered in the ED (p = 0.53) or prescribed as an outpatient (p = 0.75), incision and drainage (p = 0.19), outpatient follow-up (p = 0.53), or resolution of the symptoms (p = 0.86). However, more patients in the non-smoking group had an unplanned return to the ED as compared to those who smoked (p=0.02). In those patients who were not discharged from the ED after initial presentation, four were admitted to the hospital and 11 were transferred to a higher level of care.

Conclusion

Although drawn from a limited sample from a single rural community ED, a positive smoking history was more common among patients with a PTA. While there was no statistically significant difference in the overall treatment, a difference was noted for unscheduled return visits to the ED in those without a history of smoking.

## Introduction

Peritonsillar abscesses (PTA) are the most common deep space infection of the head and neck affecting adults between the ages of 20 and 40 [1-3]. Occurring in the United States at a rate of 30 cases per 100,000 persons, the treatment of PTA costs approximately $150 million annually [1-3]. Although several risk factors including a history of streptococcal tonsillitis and periodontal disease have been postulated as increasing a patient’s risk of developing a PTA, recent data suggest a positive correlation between those with a smoking history and the development of PTA [4-5].

Data from several recent retrospective and prospective trials have shown a direct correlation between a patient’s smoking history and the development of a PTA, where smokers are more likely to develop the infection as compared to non-smokers [6-10]. The increased risk for developing a PTA in smokers most likely occurs due to multiple processes: alteration of tonsillar bacterial flora, local and systemic immunological changes, and direct injury of the oropharyngeal mucosa [6-10]. Critically, as the smoking pack-years of a patient increases, the likelihood of these adverse effects leading to the development of a PTA increases.

Data on the correlation between smoking and the development of a PTA is currently limited and comes from countries other than the United States, where dedicated smoking cessation programs have proliferated [6-13]. Though a reduction in the rates of smoking has been seen nationwide, the rate of smoking in rural communities continues to be higher than in metropolitan areas and is associated with greater severity of negative health outcomes [14,15]. Due to the discrepancy in data available for geographically distinct rural areas, the authors sought to determine the prevalence of PTA between smokers and non-smokers who present to a rural community emergency department (ED) in the southwestern United States. Secondarily, the authors sought to determine the demographics, treatment, and outcomes between smokers and non-smokers with a PTA that was treated within the ED.

## Materials and methods

Setting

Kingman Regional Medical Center (KRMC) is a 235-bed rural community hospital in northern Arizona with an annual ED census of approximately 55,000 patient visits. The hospital is designated as a level 3 trauma center, oversees an Accreditation Council for Graduate Medical Education emergency medicine residency, and serves hospital district 1 of Mohave county. 

Protocol 

Following institutional review board approval, a retrospective chart review of all patients undergoing a soft tissue neck computed tomography (CT) with or without intravenous contrast was completed from September 25th, 2019 through October 4th, 2021. Patients were included in the study if a soft tissue neck CT was obtained within the ED that contained radiologic evidence of a PTA. Patients were excluded from the study if (1) a soft tissue neck CT was obtained outside of the ED, (2) no PTA was visualized on imaging, (3) PTA was diagnosed by some other means (ultrasound, clinical examination, etc), or (4) the patient had a previous history of PTA. All reported data were abstracted from patient charts in the MEDITECH EXPANSE Platform (Medical Information Technology Inc., Westwood, MA). Abstracted data included: demographics, imaging findings, treatments, hospitalizations, in-hospital length of stay, follow-up appointments, and outcomes. All data were abstracted by a research assistant who was blinded to the study’s objectives. The research assistant was trained on proper data abstraction prior to the collection of data by the study team. With adherence to a quality-controlled protocol and structured abstraction tool, the research assistant manually collected the remaining data points. Abstractor monitoring and verification of the independent variables were completed by the primary investigator. Those with incomplete data were removed from the final analysis. 

Statistical analysis

Data were analyzed using Statistical Product and Service Solutions (SPSS), v. 27 (IBM Corp., Armonk, NY). Patient demographics and outcomes were reported with descriptive statistics. Categorical variables were assessed with a chi-square analysis, and continuous variables were assessed with the Mann-Whitney U test. Statistical significance was defined as P ≤ 0.05.

## Results

During the time period studied, a total of 511 patients were evaluated by CT imaging in the ED for a suspected soft-tissue neck infection. Of those undergoing evaluation, 50 were diagnosed with a peritonsillar abscess by radiologic findings. The median age of those diagnosed with a PTA was 40.5 (25.5 - 53.3) years old with 30% (15/50) being female. Most patients self-identified as white, had a prior history of smoking, and self-identified as unemployed (Table 1). A total of 10 patients (seven smokers and three non-smokers) had a history of previous PTA. 

**Table 1:** Demographics, past medical history, and social history of patients diagnosed with a peritonsillar abscess following soft-tissue neck computed tomography. IQR: Interquartile range

	Total N=50	Smoking N= 27	Non-Smokers N = 23	P-Value
Age in years (IQR)	40.5 (25.5 – 53.3)	42.0 (27.0 – 50.0)	40.0 (21.0 – 59.0)	0.57
Female	15 (30%)	7 (25.9%)	8 (34.8%)	0.50
White	38 (76%)	23 (85.2%)	15 (65.2%)	0.41
Employment Status				
Employed	21 (42%)	11 (40.7%)	10 (43.5%)	0.90
Unemployed	23 (46%)	14 (51.9%)	9 (39.1%)	0.50
Retired	3 (6%)	1 (3.7%)	2 (8.7%)	0.49
Unknown	3 (6%)	1 (3.7%)	2 (8.7%)	0.49
Past Medical History				
HTN	12 (24%)	6 (22.2%)	6 (26.1%)	0.75
Heart Disease	8 (16%)	7 (25.9%)	1 (4.3%)	0.04
Diabetes	6 (12%)	4 (14.8%)	2 (8.7%)	0.51
Cancer	4 (8%)	2 (7.4%)	2 (8.7%)	0.87
Obesity	16 (32%)	9 (33.3%)	7 (30.4%)	0.55
Social History				
Alcohol	18 (36%)	10 (37.0%)	8 (34.8%)	0.87
Illicit Drugs	11 (22%)	8 (29.6%)	3 (13.0%)	0.16

In the total cohort, the median number of symptomatic days prior to presenting to the ED was 3.0 (1.75 - 5.0) days. (Table 2) Those with a smoking history presented earlier in the course of their illness as compared to those without a smoking history (2.0 vs 4.0 days; p=0.03). In the total cohort, the median diameter of the abscess was 1.75 (1.4 - 2.45) cm. No difference in the PTA diameter was noted between smokers and non-smokers (1.5 vs 2.0 cm; p=0.13). In those admitted either admitted to the hospital or transferred the median size of the PTA was 2.1 cm (1.5 - 3.8) and for those discharged from the ED 1.7 cm (1.4 - 2.3) (p=0.18). 

**Table 2:** Differences in presenting time and peritonsillar abscess size between non-smokers and smokers PTA: peritonsillar abscess; cm: centimeter; IQR: interquartile range

	Total N=50	Non-Smokers N=23	Smokers N=27	P-Value
Days to presentation (IQR)	3.0 (1.75-5.0)	4.0 (3.0 – 7.0)	2.0 (1.0 – 4.0)	0.03
PTA size in cm (IQR)	1.75 (1.4 - 2.45)	2.0 (1.5 – 2.8)	1.5 (1.4 – 2.4)	0.13

The majority of patients 74% (37/50) received a corticosteroid while in the ED, with decadron 10mg intravenously being the most common (56% (28/50) (Table 3). However, only a minority of patients received prescription corticosteroids upon discharge (24% (12/50) with the majority receiving a five-day course of prednisone 20 mg twice daily (25%; 3/12). No difference between receiving corticosteroids either while in the ED (p = 0.53) or upon discharge (p = 0.75) was found between smokers and non-smokers. A total of 24% (12/50) of patients underwent ENT consult within the ED with no difference noted between smokers and non-smokers (p = 0.73). A total of 26% (13/50) patients underwent PTA drainage within the ED with the majority receiving needle aspiration (92.3%; 12/13). No difference was noted between smokers and non-smokers (p = 0.19) in regards to drainage within the emergency department. 

**Table 3:** Differences in treatment between non-smokers and smokers with a peritonsillar abscess ED: emergency department; ENT: otorhinolaryngology: PTA: peritonsillar abscess

	Total N=50	Non-Smokers N=23	Smokers N=27	P-Value
Corticosteroid in ED	37 (74%)	18 (78.3%)	19 (70.4%)	0.53
Prescription corticosteroid	12 (24%)	6 (26.1%)	6 (22.2%)	0.75
ENT Consult in ED	12(24%)	5 (21.7%)	7 (25.9%)	0.73
PTA drainage in ED	13 (26%)	8 (34.8%)	5 (18.5%)	0.19

All patients discharged from the ED were recommended to have an outpatient follow-up scheduled. No difference was noted in discharge rates of either smokers or non-smokers (p = 0.95). Of those discharged, only a minority (20% (7/35)) followed up with ENT despite the discharge instructions (Table 4). No difference was noted between smokers and non-smokers for follow-up care (p = 0.53) nor resolution of symptoms at follow-up (p = 0.86). A total of four patients had an unscheduled return visit to the ED with all patients falling into the non-smoking category (p=0.02). No patients were transferred or admitted after representation to the ED. 

**Table 4:** Differences in follow up between non-smokers and smokers with a peritonsillar abscess ED: emergency department; ENT: Otorhinolaryngology

	Total N=35	Non-Smokers N=16	Smokers N=19	P-Value
Completed ENT follow up	7 (20%)	4 (25.0%)	3 (15.8%)	0.53
Unscheduled ED return	4 (11.43%)	0 (0%)	4 (21.1%)	0.02

In those patients who were not discharged from the ED after initial presentation, four were admitted to the hospital and 11 were transferred to a higher level of care. All patients admitted to the hospital had a smoking history and none underwent surgical drainage while admitted. In those who were transferred to a higher level of care, seven were smokers and four were non-smokers. 

## Discussion

In the current study from a rural community emergency department, 54% of PTA diagnosed by CT imaging were found in those with an active smoking history. Although those with smoking history were more likely to have a PTA as compared to non-smokers, this could be due to the relatively low incidence rate of PTA found on CT imaging. Much like the previous literature on the topic from across the world, it remains unclear if smoking predisposes a patient to the development of a PTA in a rural setting [6-13]. Larger studies examining a diverse patient population will be needed to further draw a correlation between smoking and PTA formation. 

Treatment modalities including antibiotics, corticosteroids, and surgical drainage, were similar amongst the two groups. Few patients underwent all three treatment modalities at the initial presentation despite their proven benefits in either shortening recovery time or decreasing complication rates [16]. This data is also similar to the previous literature on the topic where few patients undergo surgical management despite being given corticosteroids and antibiotics [14]. This could be due to either the lack of experience of the operator or patient preference upon discussion and shared decision making. Further studies will need to be completed in order to ascertain the main reason why few patients undergo surgical management of PTA. 

A large number of patients were either admitted to the hospital or transferred to a higher level of care for medical and surgical management. However, the overall hospitalization rate was similar between smokers and non-smokers. Although statistically significant, this is most likely due to the overall size of a PTA being larger in those admitted to the hospital as compared to those being discharged from the ED and not a patient's smoking history. 

The current study also shows that non-smokers unexpectedly represent to the emergency department more frequently than smokers despite similar treatment modalities both within the ED and as an outpatient. This is most likely due to those with a smoking history presenting and receiving treatment earlier in the course of the illness as compared to non-smokers. Much like the previous literature noting that the complication rate following treatment is rare, no patients needed admission following a second emergency department visit, and the majority seen during follow-up noted complete resolution in their symptomology. 

Limitations

One major limitation of the study is the relatively small sample size of the final analysis. Current associations seen could be altered by a larger sample size. Due to the geographic location and relatively homogenous patient population served by KRMC, data may not be generalizable to all rural community hospitals across the nation. Although KRMC serves a large portion of Mohave county, patients may have sought follow-up care or experienced unexpected ED returns at other healthcare facilities. Only patients undergoing soft tissue neck CT were included in the final results. Including other means of diagnosis such as ultrasound, point of care ultrasound, clinical suspicion, needle aspiration, and incision and drainage may have altered the results. Lastly, all demographic data including social history was self-reported by the patient. Although social history is confirmed by nursing and physicians at the given facility, data may have been altered if the patient did not include all aspects of their social history to their healthcare provider. 

## Conclusions

In this limited sample from a rural ED, the prevalence of smoking was common in those with a PTA. Even though there were no differences in the treatment between smokers and non-smokers with a PTA, smoking was associated with a shorter time interval between onset of symptoms and arrival to the ED. In those without a history of smoking, a difference was noted in return visits to the ED and could be related to the earlier treatment of smokers as compared to those who do not smoke.
